# Intergenerational effects of preconception opioids on glucose homeostasis and hepatic transcription in adult male rats

**DOI:** 10.1038/s41598-022-05528-w

**Published:** 2022-01-31

**Authors:** Anika M. Toorie, Fair M. Vassoler, Fangfang Qu, Donna Slonim, Christopher M. Schonhoff, Elizabeth M. Byrnes

**Affiliations:** 1 Department of Biomedical Sciences, Cummings School of Veterinary Medicine, Tufts University, 200 Westboro Road, Peabody Pavilion, North Grafton, MA USA; 2 Department of Computer Sciences, School of Arts and Sciences, Tufts University, Medford, MA USA

**Keywords:** Physiology, Systems biology, Diseases, Endocrinology

## Abstract

Adolescence represents a period of significant neurodevelopment during which adverse experiences can lead to prolonged effects on disease vulnerability, including effects that can impact future offspring. Adolescence is a common period for the initiation of drug use, including the use of opioids. Beyond effects on central reward, opioids also impact glucose metabolism, which can impact the risk of diabetes. Moreover, recent animal models suggest that the effects of adolescent opioids can effect glucose metabolism in future offspring. Indeed, we demonstrated that the adult male offspring of females exposed to morphine for 10 days during adolescence (referred to as MORF1 males) are predisposed to the adverse effects of an obesogenic diet. As adults, MORF1 males fed a high fat moderate sucrose diet (FSD) for just 6 weeks had increased fasting glucose and insulin levels when compared to age-matched offspring of females exposed to saline during adolescence (SALF1 males). Clinically, a similar profile of impaired fasting glucose has been associated with hepatic insulin resistance and an increased risk of non-alcoholic fatty liver disease. Thus, in the current study, we used RNA sequencing to determine whether adult MORF1 males demonstrate significant alterations in the hepatic transcriptome suggestive of alterations in metabolism. Age-matched SALF1 and MORF1 males were fed either FSD or control diet (CD) for 8 weeks. Similar to our previous observations, FSD-maintained MORF1 males gained more weight and displayed both fasting hyperglycemia and hyperinsulinemia when compared to FSD-maintained SALF1 males, with no significant effect on glucagon. No differences in bodyweight or fasting-induce glucose were observed in control diet (CD)-maintained F1 males, although there was a trend for CD MORF1 males to display elevated levels of fasting insulin. Unexpectedly, transcriptional analyses revealed profound differences in the hepatic transcriptome of CD-maintained MORF1 and SALF1 (1686 differentially expressed genes) with no significant differences between FSD-maintained MORF1 and SALF1 males. As changes in the hepatic transcriptome were not revealed under 8 weeks FSD conditions, we extended the feeding paradigm and conducted a glucose tolerance test to determine whether impaired fasting glucose observed in FSD MORF1 males was due to peripheral insulin resistance. Impaired glucose tolerance was observed in both CD and FSD MORF1 males, and to a more limited extent in FSD SALF1 males. These findings implicate intergenerational effects of adolescent morphine exposure on the risk of developing insulin resistance and associated comorbidities, even in the absence of an obesogenic diet.

## Introduction

Despite efforts to decrease opioid prescription rates, use and misuse of prescription opioids among adolescents’ remains higher than that observed in previous decades^1–3^. Given the important role of endogenous opioids in regulation of multiple homeostatic processes, including regulation of both the hypothalamic pituitary adrenal (HPA) and the hypothalamic pituitary gonadal (HPG) axes^4,5^, exogenous opioids during this critical period may have significant long-term effects. The developmental origin of health and disease (DOHAD) best encapsulates the notion of epigenetic inheritance of disease, including epigenetic mediated metabolic programming, where an experience in the F0 generation results in an altered metabolic state in F1 progeny and possibly beyond^6^. An association between F0 nutritional status and metabolic programming has been the focus of many studies; however, there is limited information regarding the impact of F0 opioid exposure on offspring metabolic programming. Drugs of abuse, including opioids, can impair glucose homeostasis. Clinically, opioids diminish insulin peak time and glucose clearance in affected patients. Furthermore, there is an association between opioid-induced fasting hyperinsulinemia, even when occurring in the absence of obesity, and glucose intolerance. Thus, there is an established relationship between direct opioid use and gross glucose dysregulation due to alterations in endocrine glucoregulatory hormones^7–11^. Recently, studies have started to explore the multigenerational consequences of opioid-induced metabolic programming. Previous findings demonstrate that adolescent opioid exposure can have significant endocrine effects in both males and females^12,13^; effects that could impact the development of their future progeny. Indeed, we and others have previously documented significant effects of adolescent morphine exposure in both males and females on future offspring, with some effects observed in both F1 and F2 generations^14–21^. These effects are not due to direct fetal exposure or to effects of acute withdrawal, as exposed animals are mated several weeks after their final dose of morphine. While the mechanism of transmission remains to be determined, inter- and transgenerational effects of adolescent opioid exposure in rodents have now been well established.

Many of the previous studies investigating intergenerational effects of drugs of abuse have largely focused on neuronal modifications that can impact abuse liability in adult offspring^14–16^. Next-generation effects of parental opioid-exposure included sex-specific alterations in stress-axis physiology, neurobehavioral responses of reward and motivation, and development. Recently, we initiated a series of experiments examining the effects of adolescent opioids on metabolic homeostasis in F1 progeny^17^. These studies were based on the prominent role endogenous opioids, such as the proopiomelanocortin (POMC)-derived β-endorphin, play in the regulation of energy balance^18–20^. We examined F1 progeny under different dietary conditions, with rats either fed a high fat/moderate sugar diet (FSD) or a control diet (CD). After only 6 weeks, FSD maintained male offspring of adolescent morphine exposure females (MORF1) displayed positive energy balance, impaired fasting glucose, and modifications in their glucoregulatory endocrine profile when compared to offspring of adolescent saline exposed females (SALF1) fed the same diet^22^. Metabolic effects were not observed in CD-maintained F1 males, although even under CD conditions, MORF1 males displayed delayed pubertal development and elevated levels of POMC peptide in the arcuate nucleus^22^.

**Figure 1:**
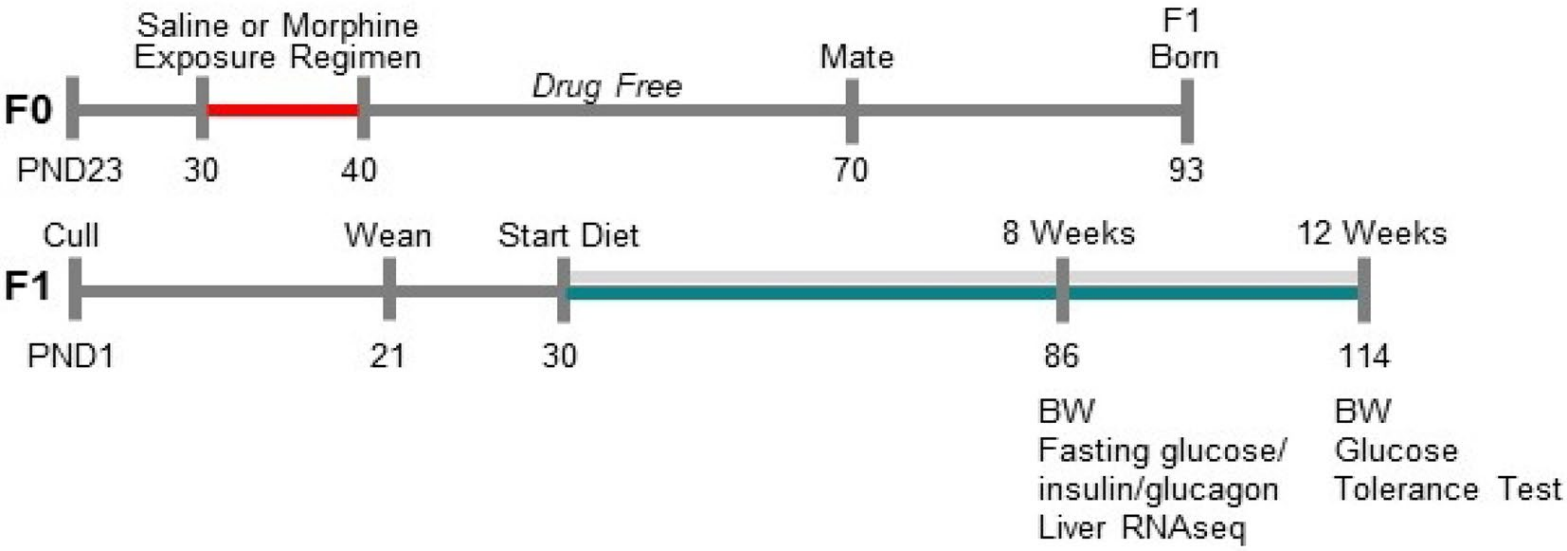
Schematic of experimental design.

Numerous studies implicate POMC-derived neuropeptides in both hypothalamic and brainstem nuclei as important mediators of energy homeostasis^23^; however, the observed pre-diabetic condition displayed in FSD-maintained MORF1 males under fasting conditions suggests the emergence of insulin resistance. Clinically, impaired fasting glucose is associated with significant deficits in hepatic glucose regulation, including hepatic insulin resistance^24,25^. Thus, in the current study, we conducted hepatic RNA sequencing under fasting conditions in both CD and FSD maintained F1 males to test the hypothesis that MORF1 males exhibited alterations in their hepatic transcriptome relative to SALF1 males; thereby contributing to glucose dyshomeostasis, an effect we conjectured to be further amplified by chronic FSD conditions. A schematic of our experimental design is shown in Fig. 1. Following 8-weeks of diet maintenance, significant differences in F1 hepatic gene transcription were observed in CD-maintained males, despite similar levels of 24-h fasting glucose. Therefore, we conducted glucose tolerance testing to determine whether CD-fed F1 males demonstrated impaired glucose clearance following 12-weeks of diet maintenance. Overall, our findings implicate significant intergenerational effects of limited female adolescent morphine exposure on glucose homeostasis, even in the absence of an obesogenic diet. Yet, our findings also suggest that the negative effects of long-term FSD feeding are amplified as a consequence of maternal F0-morphine exposure. Thus, collectively our findings suggests that female adolescent opioid exposure is sufficient to induce intergenerational metabolic programming in F1 male offspring.

## Results

**Figure 2:**
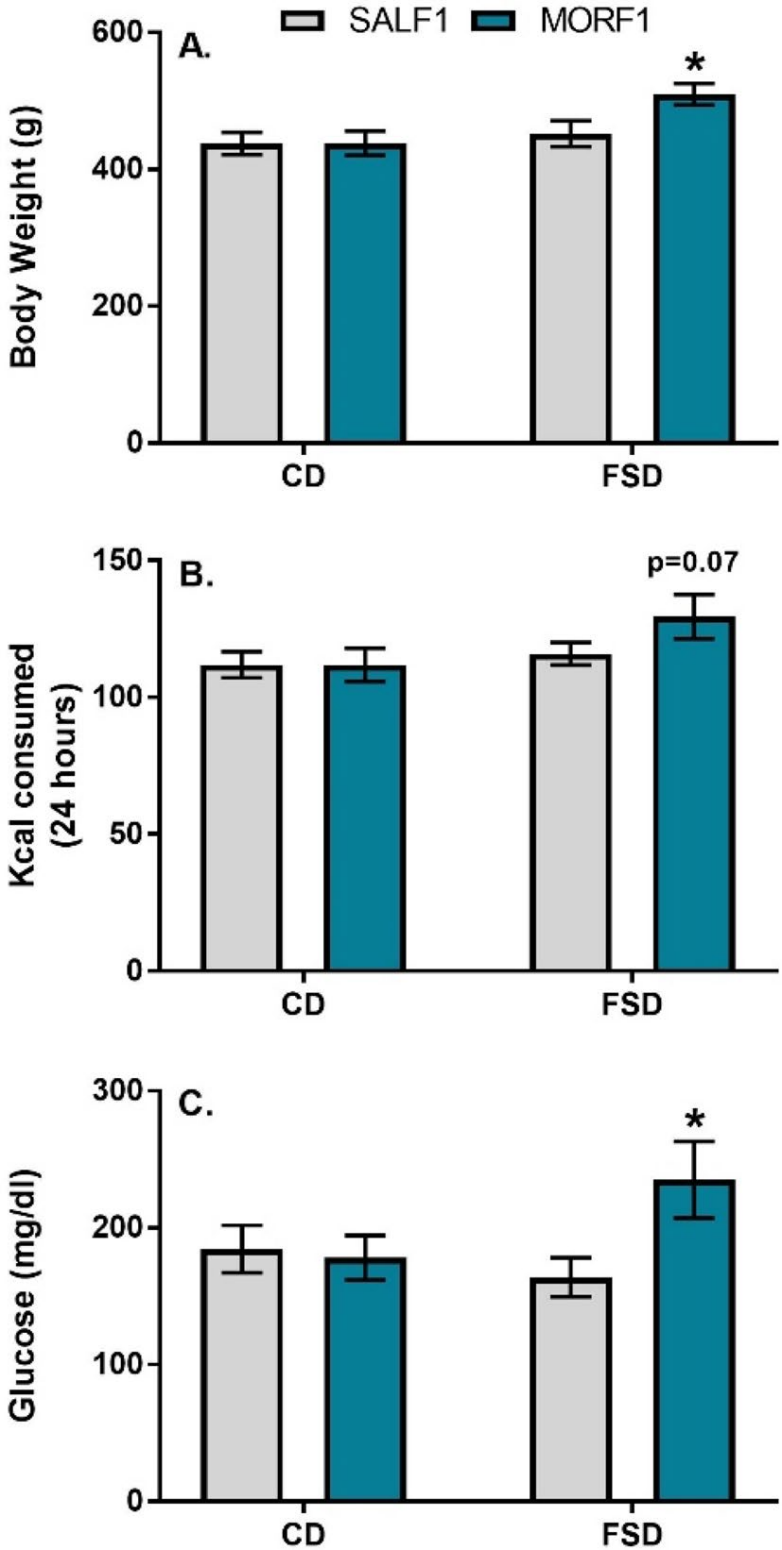
Panel (**A**): Mean (± SEM) bodyweight after 8 weeks on diet; N = 9/group. Panel (**B**): Mean (± SEM) food consumption in kilocalories over a 24 h period after 8 weeks on diet; N = 9/group Panel (**C**): Mean (± SEM) blood glucose following a 24 h fast; N = 9 for all groups but SALF1 FSD with N = 8 due to removal of statistical outlier; *p < 0.05 as compared to SALF1 FSD.

As shown in Fig. 2 (panel A), similar to our previous findings, after 8 weeks, FSD-maintained MORF1 males were significantly heavier than their SALF1 counterparts. Two-way ANOVA revealed a significant main effect of F1 diet (F_(1,32)_ = 6.2; *p* = 0.02), with no main effect of F0 adolescent exposure F_(1,32)_ = 2.8; *p* = 0.1, and no significant interaction (F_(1,32)_ = 2.8; *p* = 0.1). Based on our prior findings, we conducted an a priori *t* test within the FSD condition, which revealed a significant effect in MORF1 males (t_[15]_ = 2.3; *p* = 0.03). No significant differences were observed with regard to 24 h food intake (main effect of F0 adolescent exposure F = _(1,32)_ = 1.3; *p* = 0.26; interaction F = _(1,32)_ = 1.3; *p* = 0.25), although there was a trend toward a main effect of F1 diet (F_(1,32)_ = 3.3; *p* = 0.07) on food intake, which appeared to be due to a non-significant (*p* = 0.07) increase in calories consumed by FSD MORF1 males (see Fig. 2; panel B). Following a 24 h fast, these same animals were monitored for blood glucose levels. One statistical outlier was removed from SALF1 FSD group. As shown in Fig. 2 (panel C), while there were no main effects of either F1 diet (F_(1,30)_ = 0.81; *p* = 0.37) or F0 adolescent exposure (F_(1,30)_ = 2.56; *p* = 0.11), there was a trend toward an interaction (F_(1,30)_ = 3.67; *p* = 0.06). Again, based on our previous findings, we conducted an a priori *t* test in FSD-maintained males, which revealed significantly higher levels of glucose in FSD MORF1 males (t_[15]_ = 2.17; *p* = 0.04).

**Figure 3:**
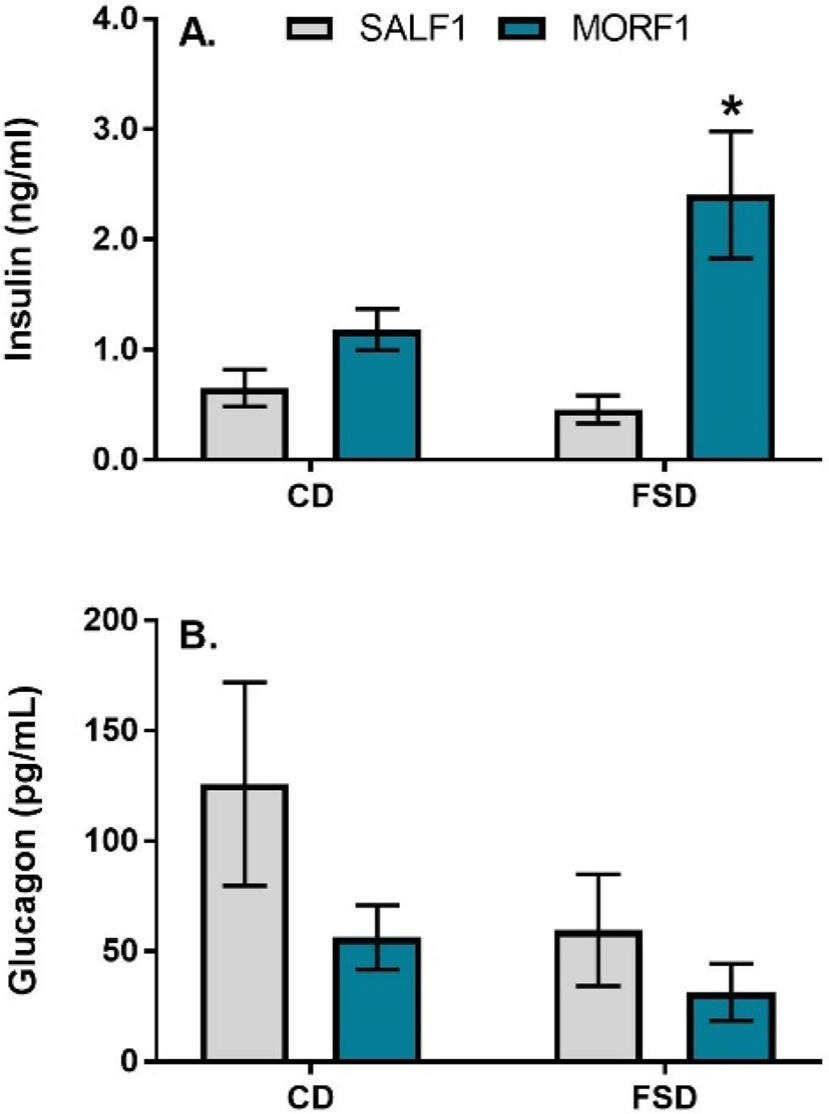
Panel (**A**): Mean (± SEM) plasma insulin levels (ng/ml) following a 24 h fast; SALF1 CD N = 6; SALF1 FSD N = 5; MORF1 CD N = 5; MORF1 FSD N = 6; *p < 0.05 as compared to SALF1 FSD. Panel (**B**): Mean (± SEM) plasma glucagon levels (pg/ml) following a 24 h fast; SALF1 CD N = 8; SALF1 FSD N = 9; MORF1 CD N = 6; MORF1 FSD N = 8.

As illustrated in Fig. 3, we also examined insulin and glucagon following the 24 h fast. For insulin (panel A), there was a significant main effect of adolescent exposure (F_(1,18)_ = 12.6; *p* = 0.002), no main effect of diet (F_(1,18)_ = 2.1; *p* = 0.15) and a marginally significant interaction (F_(1,18)_ = 4.1; *p* = 0.05). These effects were due to significantly elevated insulin levels in FSD-maintained MORF1 males when compared to FSD SALF1 males (*p* < 0.05). For glucagon (Fig. 3; panel B), there were no significant effects of adolescent treatment (F_(1,27)_ = 2.6; *p* = 0.11) or diet (F_(1,27)_ = 2.3; *p* = 0.13) and no interaction (F_(1,27)_ = 0.4; *p* = 0.49).

**Figure 4:**
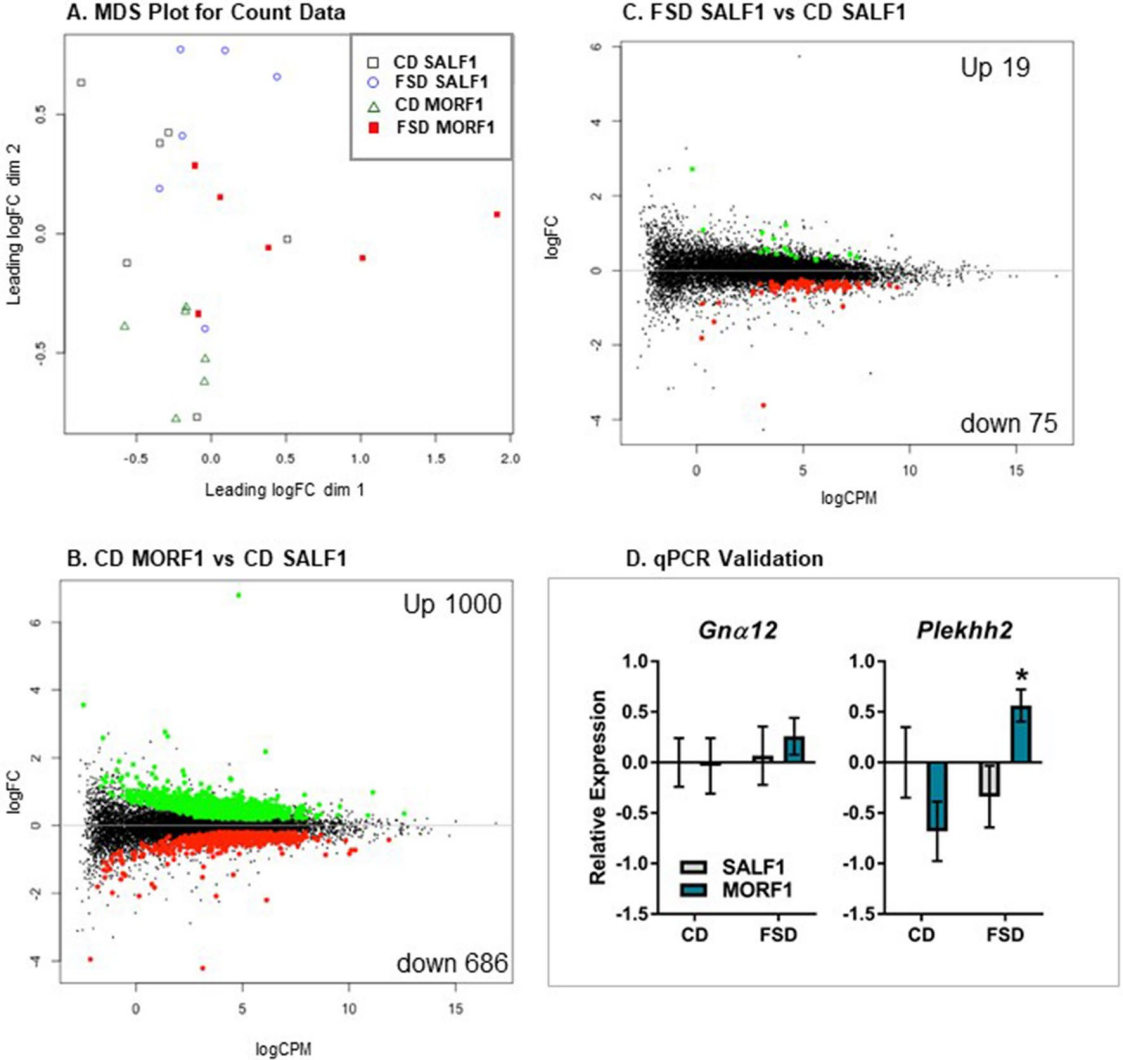
Panel (**A**): MDS Plot comparing all groups. Panel (**B**): Volcano plot of significant differential gene expression when comparing CD maintained MORF1 and SALF1 males. Panel (**C**): V Volcano plot of significant differential gene expression when comparing FSD maintained SALF1 and CD maintained SALF1 males. Panels (**A**–**C**) N = 6/group. Relative expression of Gnα12 (top) and Plekhh2 (bottom) in punch biopsies of liver samples taken after a 24 h fast. SALF1 CD N = 6; SALF1 FSD N = 6; MORF1 CD N = 7; MORF1 FSD N = 7; *p < 0.05 as compared to all other groups.

To identify potential changes in the liver associated with the increased fasting glucose and insulin levels observed in FSD MORF1 males, we conducted hepatic RNA sequencing. Library size ranged from 15 to 20 million reads. A minimal filtering method was employed (minimum count of ≥ 10 in at least 6 samples) which resulted in 13,405 genes included in the analyses. Differential gene expression with a *q* value of < 0.05 was used to determine main effects of F0 adolescent exposure and diet as well as their interaction. As shown in Fig. 4A, when multidimensional scaling (MDS) was applied to visualize the relatedness of individual subjects, CD maintained MORF1 males formed a distinct cluster with clear separation from CD maintained SALF1, while FSD maintained animals demonstrated greater variability.

Statistical analyses of transcriptomic data revealed significant differences based on both F0 adolescent exposure and diet condition. As reported in Table 1, F0 adolescent morphine exposure was associated with 724 differentially expressed hepatic genes, while FSD maintenance was associated with 129 differentially expressed genes (main effects). Unexpectedly, when comparing the effects of F0 adolescent exposure within FSD maintained animals, there were no significant differences in hepatic gene expression. Yet, in CD maintained animals, F0 adolescent exposure led to 1686 differentially expressed genes as shown in Fig. 4B. These findings suggest the effects of F0 adolescent morphine exposure on F1 male progeny precede the metabolic effects of FSD. When examining the effects of FSD within MORF1 progeny, only 1 gene, *Gna12 (G Protein Subunit α12)* was significantly down-regulated, however, that effect was not corroborated with follow up qPCR (all *p*’s > 0.4; Fig. 4D; top panel). In contrast, maintenance on FSD in SALF1 males resulted in 94 differentially expressed genes when compared to SALF1 CD males (see Fig. 4C). Finally, only a single gene, *Plekhh2* (pleckstrin homology domain containing, family H (with MyTH4 domain) member 2), which codes for a protein involved in actin dynamics, demonstrated a significant interaction. *Plekhh2* was significantly upregulated in MORF1 FSD males compared to all other groups, an effect that was validated in follow up qPCR samples (see Fig. 4D; bottom panel). Complete gene lists are available in Supplemental File 1.

**Table 1:** Number of differentially expressed genes that reach significance comparing F0 treatment x dietary condition.

Comparison	Up	Down	Total
Main effect of F0 treatment	415	309	724
Effect of F0 treatment CD fed	1000	686	1686
Effect of F0 treatment FSD fed	0	0	0
Main effect of diet	41	88	129
Effect of FSD in SALF1	19	75	94
Effect of FSD in MORF1	0	1	1
F0 treatment × diet Interaction	1	0	1

q value < 0.05.

Given the number of genes demonstrating significant differences, we examined enriched pathways in this data set using Gene Ontology (GO) enrichment with g:Profiler^26^. When analysis was restricted to manually curated evidence (i.e. no IEA annotations), pathways up-regulated in MORF1 CD males (as compared to SALF1 CD males) largely identified pathways involved in epigenetic regulation of transcription. These pathways include GO Molecular Functions such as *ubiquitin-specific protease binding*, GO Biological Process terms such as *histone H4-K16 acetylation*, and GO Cellular Component terms, such as RISC-loading complex. Genes downregulated in CD MORF1 males were enriched for terms related to cellular respiration. These pathways include GO Molecular Functions such as *electron transfer activity*, GO Biological Process terms such as *mitochondrial respiratory chain complex assembly,* and GO Cellular Component terms related to mitochondria and ribosomes (see Supplemental File 2 for complete list of significant GO enrichment terms). Together, these findings suggest that maternal opioid exposure prior to conception leads to epigenetic modifications and reduced cellular respiration within the liver of CD maintained males despite the absence of significant effects on bodyweight or fasting glucose.

Gene sets enriched in FSD SALF1 males as compared to CD SALF1 males, also implicate enhanced transcriptional activity and diminished cellular respiration, although these effects are more muted than the effects of F0 adolescent exposure. Gene terms upregulated by FSD in SALF1 males include only one GO Molecular Function, *UDP-N-acetylglucosamine diphosphorylase activity*, an enzymatic process involved in amino sugar metabolism. No significant effects of FSD on GO Biological Processes or Cellular Component terms were identified. Gene sets downregulated by FSD in SALF1 males identified GO Molecular Function terms involving electron transport chain enzymes, GO biological processes associated with cellular respiration, and GO Cellular Component terms associated with mitochondria (see Supplemental File 2). Overall, these findings suggest reduced efficiency in supporting the fasting-induced energy requirements of hepatocytes following maintenance on FSD in SALF1 males.

**Figure 5:**
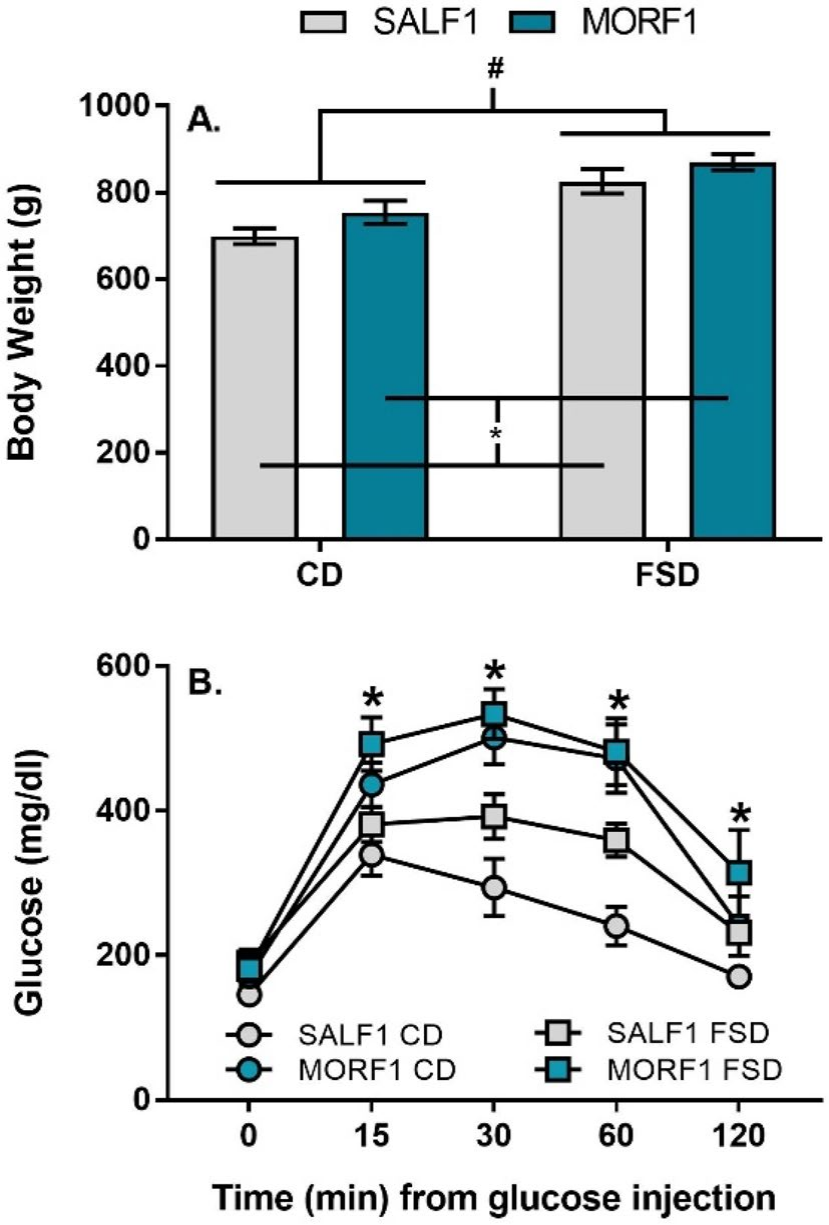
Panel (**A**): Mean (± SEM) bodyweight after 12 weeks on diet; #*p* < 0.05 main effect of diet; *p < 0.05 main effect of F0 morphine exposure; SALF1 CD N = 10; SALF1 FSD N = 10; MORF1 CD N = 8; MORF1 FSD N = 8. Panel (**B**): Mean (± SEM) blood glucose level in response to a glucose challenge in CD or FSD males; *p < 0.05 as compared SALF1 males maintained on the same diet; SALF1 CD N = 10; SALF1 FSD N = 10; MORF1 CD N = 8; MORF1 FSD N = 8.

Overall, the significant hepatic transcriptional modifications observed in CD MORF1 males under fasting conditions suggest that despite the absence of differences in body weight and normal fasting glucose levels, these animals may be metabolically challenged. To further explore this possibility, a separate set of CD and FSD F1 males was generated and maintained on their respective diets for 12 weeks. What emerged with more prolonged exposure in these subjects was a main effect of both F0 adolescent morphine exposure (F_(1,32)_ = 4.433; *p* = 0.04) and diet on body weight (F_(1,32)_ = 26.1; *p* < 0.001) with no interaction (*p* = 0.8), as shown in Fig. 5A. In response to a glucose challenge, both CD and FSD MORF1 males demonstrate impaired glucose clearance. Thus, there was a main effect of both F0 adolescent morphine exposure (F_(1,28)_ = 22; *p* < 0.001) and diet on glucose level (F_(1,28)_ = 5.9; *p* = 0.02) with no interaction (*p* = 0.4). When examined over time, there was a significant within-subject effect of time by F0 adolescent morphine exposure (F_(4,112)_ = 5.5; *p* < 0.001) but no time by diet (*p* = 0.7) nor time by F0 adolescent morphine exposure by diet effects (*p* = 0.4). These effects, which are illustrated in Fig. 5B, demonstrate significantly delayed glucose clearance in MORF1 males when compared to SALF1 males, regardless of dietary condition (all *p*’s < 0.01).

## Discussion

The current set of findings corroborates our previous work regarding intergenerational effects of adolescent morphine exposure on metabolism. Despite the absence of any direct morphine exposure in the developing F1 fetus, as adults, MORF1 males demonstrate metabolic dyshomeostasis suggestive of metabolic syndrome and emerging type 2 diabetes. These effects are coincident with marked effects on hepatic gene expression under fasting conditions even in the absence of any exposure to an obesogenic diet. The pattern of effects suggests the emergence of both hepatic and peripheral insulin insensitivity.

We previously observed increased body weight gain accompanied by fasting-induced hyperglycemia, hyperinsulinemia and hypercorticosteronemia in MORF1 males following FSD maintenance for 6 weeks^27^. Together, these findings suggested an increased liability of diabetes in response to dietary challenge in MORF1 males. In the current set of studies, subjects were FSD maintained for either 8 (fasting-induced effects and liver sequencing) or 12 weeks (glucose clearance) to further understand the impact of an obesogenic challenge on glucose homeostasis in MORF1 males. After 8 weeks, similar effects were observed with regard to body weight and glucose with MORF1 FSD males heavier than their SALF1 FSD counterparts and with increased levels of fasting glucose. We also examined caloric consumption to reconcile changes in bodyweight, as we previously observed increased caloric consumption in MORF1 FSD males with no change in locomotor activity^27^. Changes in food intake in the current study were not significant, although MORF1 FSD males still tended to demonstrate increased consumption. While locomotor activity was not evaluated in the present study, it is possible that one or more mechanism contributing to energy expenditure was negatively impacted in MORF1 FSD, thus contributing to weight gain. Indeed, Lu et al., recently reported that 8 weeks of high fat diet (HFD) feeding resulted in impaired mitochondrial respiratory function concurrent with dyslipidemia that worsened as the feeding period was extended. Further, while HFD fed males maintained normal glycemia following the 8-week feeding paradigm, prolonged feeding (20 weeks) was sufficient to induce hyperglycemia^28^.

We did replicate effects on fasting-induced insulin in MORF1 FSD animals; however, in our previous findings there was no main effect of F0 exposure or diet on fasting-insulin in CD F1 males, with the effect driven solely by increased insulin in FSD MORF1 males. In the current set of findings, while fasting insulin levels in MORF1 CD males were not significantly higher than SALF1 CD males, there was a trend toward increased insulin (*p* = 0.07), suggesting that insulin insensitivity may begin to emerge over time in MORF1 males, even in the absence of FSD. Indeed, after 12 weeks diet maintenance, glucose clearance was significantly impaired in MORF1 FSD and CD males. The precise time course for the emergence of these effects remains to be determined but it is clear that while FSD maintenance expedites the emergence of metabolic dyshomeostasis, F0 adolescent exposure in females is sufficient to induce next generation metabolic effects, even in the absence of an obesogenic diet.

While a significant effect of F0 adolescent exposure on fasting insulin levels was observed, no significant effects on glucagon were detected. Similar findings have been reported in clinical populations with no change in insulin-mediated suppression of glucagon observed in populations with fasting hyperglycemia, impaired glucose tolerance, or type 2 diabetes^29^. Thus, it is unlikely that glucagon-mediated stimulation of liver gluconeogenesis plays a primary role in the observed hyperglycemia in FSD MORF1 males. In contrast, the fasting stimulated increase in glucagon displayed by SALF1-CD animals may account for the similar levels of fasting glycemia observed between SALF1 CD and FSD males. Indeed, a chronic fast was shown to upregulate circulating glucagon and corticosterone in an effort to resolve hypoglycemia^30^, while chronic maintenance on an obesogenic diet is known to dysregulate glucagon secretion under fasting conditions^31^. Our current and prior endocrine (glucagon, insulin, and corticosterone) data suggests that a maternal history of opioid exposure induced a dysregulated glucoregulatory profile, which was further exacerbated by chronic FSD. Moreover, MORF1 males exhibited delayed sexual maturation in comparison to SALF1 males^27^, which may highlight an underlying dysfunction of the hypophysiotropic gonadal (HPG) axis. In male humans, dysregulated HPG activity resulting in hypogonadism can induce insulin resistance in a proportion of affected individuals, occurring alongside normoglycemia. Furthermore, metabolomic plasma comparison of insulin resistant (IR) or insulin sensitive hypogonadic males revealed that those with IR exhibited diminished expression of genes involved in cellular respiratory pathways, occurring in tandem with upregulated amino acid degradation, aimed at stimulating hepatic gluconeogenesis via amino acid conversion to glycolytic precursors^32^. Collectively, our endocrine and developmental findings imply a broad impact on endocrine systems that influence glucose homeostasis in MORF1 males.

Impaired fasting glucose with hyperinsulinemia is suggestive of hepatic insulin resistance. Yet, when examining F1 FSD males, no significant differences in hepatic transcription were revealed. It is possible that F0 adolescent exposure and FSD lead to alterations in common pathways regulating intracellular bioenergetics, essentially dampening differences in gene expression between FSD maintained SALF1 and MORF1 males. In contrast, differential gene expression when comparing CD F1 males suggests profound differences in hepatic gene transcription in the progeny of F0 adolescent morphine exposed females, despite their similar body weights and fasting glucose levels. Based on KEGG functional pathway analysis^33^ the dominant term impacted in CD MORF1 males is *Ribosome* (see Supplemental File 3). Yet, with the exception of the *Ribosome* term, the other top terms altered by F0 adolescent morphine exposure under CD conditions are similar as those affected by FSD in SALF1 males (e.g. *Non-alcoholic fatty liver disease*, *oxidative phosphorylation, diabetic cardiomyopathy, Alzheimer’s disease*; see Supplemental File 3). This suggests that F0 adolescent morphine exposure, even under CD conditions, results in alterations in hepatic transcription consistent with the effects of FSD maintenance^28^. Moreover, many of the pathways identified implicate metabolic and inflammatory processes that are frequently associated with negative health outcomes. Together, our prior and current transcriptome findings suggest that male MORF1s are primed towards development of metabolic syndrome via the modulation of key hypothalamic and hepatic transcriptional networks.

The distinct category of *Ribosome* that emerges only in MORF1 CD males may provide insight into early changes in this phenotype that ultimately lead to impaired glucose clearance. It should be noted that this term includes genes associated with both cellular and mitochondrial ribosomes. Indeed, many of the genes differentially expressed in MORF1 CD males implicate changes in mitochondrial function. Such effects are intriguing given the connection between mitochondria and liver damage^34^. Moreover, the association with NAFLD may also be a key indicator. NAFLD is the result of mitochondrial dysfunction and clinical findings demonstrate that NAFLD in metabolically healthy adults is an early predictor of metabolic disease, including type 2 diabetes^35^. While we did not perform histological evaluation of hepatic tissue for morphological changes indicative of NAFLD, metabolically healthy MORF1 CD animals demonstrate upregulated transcriptional pathways associated with NAFLD and subsequently demonstrate impaired glucose clearance. Thus, CD MORF1 males appear predisposed to develop metabolic disorders, with a liver transcriptome similar to that observed following chronic exposure to an obesogenic diet. These effects may be exacerbated when MORF1 males are fed an obesogenic diet, thus enabling the emergence or progression of metabolic dysfunction. Of note, *Plekhh2* was the only gene that was significantly upregulated in FSD MORF1 males when compared to all other groups, including CD MORF1 males. While the role of *Plekhh2* in the liver is currently unknown, *Plekhh2* was implicated in G protein signaling pathways^36^ and clinically, GWAS data have linked a single nucleotide polymorphism in *Plekhh2* with new onset diabetes in response to antihypertensive medication^37^. Future studies are needed to determine whether Plekhh2 plays a role in the IFG observed in FSD MORF1 males.

Significant deficits in glucose clearance were observed in both CD and FSD MORF1 males, suggesting peripheral insulin insensitivity. Clinically, IFG and IGT are both considered signs of prediabetes but they are not always observed together, as they represent different homeostatic processes, with IFG associated with hepatic insulin insensitivity and IGT associated with peripheral insulin insensitivity. MORF1 FSD males demonstrate both IFG and IGT, while MORF1 CD males only demonstrate IGT. It is possible that differences in fasting-induced hepatic transcription in MORF1 CD males represent compensatory processes that allow for the maintenance of fasting glucose levels. Such compensatory processes may fail in those animals maintained on FSD, which then results in activation of hypothalamic transcriptional pathways in response to glucose dyshomeostasis. Of note, studies have observed that even two weeks after initiation of high fat diet, mild impairment in glucose tolerance can be observed which is coincident with deficient glucose uptake by the liver, but not by fat or muscle^38^. Such effects could be exacerbated in MORF1 males based on the differences in hepatic transcription observed in the absence of a high fat diet.

In the current set of studies, IGT was examined following an extended feeding period to allow the progression of metabolic dysfunction, with all animals tested at 114–116 days of age. There was a significant main effect of FSD on IGT, which is similar to results reported in other nutritional studies^39^. In addition, both CD and FSD MORF1 males demonstrated IGT. Whether IGT would have been observed at earlier time points remains to be determined. Following 12 weeks of dietary feeding, however, there was also a main effect on body weight, which suggests that MORF1 CD were beginning to shift towards increased weight gain. Again, additional studies are needed to determine the emergence of these effects, whether they are driven by effects on feeding behavior or energy expenditure, and to what extent they correlate with changes in glucose homeostasis. These findings, however, indicate that even in the absence of FSD, MORF1 males display a prediabetic phenotype associated with an increased risk of developing a number of metabolic disorders.

The mechanism of transmission of this phenotype, given the absence of any fetal exposure to morphine, remains unknown. Maternal/fetal effects on adult metabolism are well-described, although largely in the context of maternal diet (under- and over-nutrition), gestational diabetes, and/or placental insufficiency^40–43^. Certainly, intrauterine growth restriction is a well-known risk factor for metabolic disease^44^. More controversial, although with mounting evidence, is the concept of inter- and transgenerational epigenetic effects that can instigate and propagate metabolic phenotypes across multiple generations^45–47^. For example, while the precise mechanism of inheritance remains to be uncovered, obesogenic (endocrine disrupting) chemicals^48^ have been shown to influence adipocyte mass and hepatic steatosis in both a multigenerational and transgenerational manner. F1, F2, and F3 offspring exhibited altered expression of genes involved in hepatic lipogenesis and lipolysis^49^. Taken together, evidence support the notion of the liver as a key target of metabolic programming; however, additional studies are required to elucidate the specific epigenetic mode of transmission.

Epigenetic-mediated (i.e., N-terminal tail histone modifications, DNA methylation) gene expression changes in reward-related brain regions have been reported to accompany the neurobehavioral phenotype of F0-opioid exposed offspring and grand offspring^50^. The current study extends our understanding of the multigenerational impact to the hepatic transcriptome of the progeny of F0-opioid exposed females, as we identified global enrichment of epigenetic pathways in the hepatic tissue of MORF1 animals (i.e. histone H4-K16 acetylation). However, additional studies are needed to resolve the specific genes that are permanently altered due to a maternal history of adolescent opioid use, as well as the epigenetic mechanism mediating its mode of transcriptional transmission. Nonetheless, given the broad role of endogenous opioids with regard to both reproduction and energy homeostasis, persistent effects of F0 morphine capable of influencing the development of her future offspring are plausible. Thus, while no significant effects on maternal behavior or the gross development of F1 offspring have been observed in this paradigm^51,52^, modifications at the germline level, within the uterine environment, or in the postnatal environment (e.g. alterations in milk content) are all possible. It should also be noted that mitochondrial DNA is inherited via the maternal germline^53^ and there is evidence that morphine can significantly alter mitochondrial DNA^54^. As our findings in the liver suggest mitochondrial dysfunction in MORF1 CD males, one intriguing, yet speculative, possibility is that F0 adolescent morphine exposure triggers intergenerational effects via the inheritance of mitochondrial DNA with more limited oxidative respiratory capacity. Certainly, future studies are needed to identify what mechanism(s) underlies the transmission of increased metabolic risk to future progeny following adolescent morphine exposure. The current findings, however, support a link between preconception maternal opioid exposure and male adult onset of glucose dyshomeostasis associated with altered hepatic transcription suggestive of NAFLD.

## Methods

### Experimental design

As shown in Fig. 1, two experiments were independently run with key differences in feeding period highlighted. For both experiments, SALF1 and MORF1 males were generated as described below. Experiment 1 concluded after an 8-week feeding period, at which point animals were subjected to a 24-h fast. Experiment 2 concluded following 12-weeks diet maintenance, in order to magnify diet effects, at which point animals were subjected to a 16-h fast preceding glucose tolerance testing. For both experiments, the endpoint parameters that were later evaluated are noted.

### Adolescent morphine exposure

For all experiments 22-day-old female Sprague–Dawley rats [Crl:CD(SD)BR] were purchased from Charles River Breeding Laboratories and grouped housed in light-(on 600–1800 h) and temperature-(21–24 °C) regulated rooms with ad libitum access to food and water. All experiments were conducted with an approved protocol from the Tufts University Institutional Animal Care and Use Committee (Protocol # G2017-43) and all experiments and methods were performed in accordance with the relevant regulations and ARRIVE guidelines. Adolescent rats were randomly assigned to receive either morphine or saline beginning on postnatal day 30 (PND30) for 10 days, which corresponds to the early-mid adolescent phase in humans^55^. Morphine was subcutaneously administered between 9:00 and 10:00 AM via daily injections with an intermittent, increasing dose regimen of morphine that increased every other day (5, 10, 15, 20, 25 mg/kg); dosing was based on allometric scaling and is used to model escalating use patterns observed in humans. SALF0 females were administered USP grade saline at equivalent volumes.

### Mating

All F0 females remained drug free for at least 3 weeks after the final morphine injection and were subsequently mated as adults (after PND70) with drug-naïve colony males. On the day following parturition (PND1), all F1 litters were weighed and culled to five males and five females. As our prior findings indicated that MORF1 males exhibited hypothalamic dysregulation concurrent with glucose dyshomeostasis, these follow-up studies were restricted to male offspring. On PND21 all F1 litters were weaned and transitioned to standard rodent chow. To minimize litter confounds, only one subject per litter was used in any experimental condition. Sample sizes reported represent a single subject from a litter. A total of N = 25 SALF1 litters and N = 23 MORF1 litters were generated in three separate cohorts for these studies.

### Diets

On PND30, MORF1 and SALF1 males were randomly assigned to begin maintenance on a control diet (3.8 kcal/g) consisting of 10% fat /0%sucrose (D12450K; Research diets, New Brunswick, NJ, USA) or a high fat-moderate sucrose diet (FSD) (4.7 kcal/g) consisting of 45% fat /17% sucrose diet (D12451; Research diets) for a period of 8 weeks (N = 15 SALF1 litters and N = 15 MORF1 litters) or 12 weeks (N = 10 SALF1 litters and N = 8 MORF1).

### Bodyweight measurements

Bodyweight gain was calculated by subtracting individual PND30 bodyweights from PND86 bodyweights.

### Blood and liver collection

Animals were sacrificed between 9:00 and 10:00 AM following a 24 h fast. Rats were transferred to a procedure room (transit < 60 s), exposed briefly to CO_2_ and decapitated. The order of sacrifice was counterbalanced across all 4 groups to avoid order effect confounds. Glucose was measured immediately from trunk blood via a standard glucometer (CVS, MA). Livers were removed, rapidly frozen in − 20 °C 2-methylbutane, and stored at  − 80 °C until the time of processing.

### Glucose tolerance testing

Animals were fasted overnight (16 h). The following day (9:00–10:00 AM) a baseline blood sample was collected via tail nick. Subjects were then injected with d-glucose (2 mg/kg, i.p) and blood was collected by tail nick at 15, 30, 60, and 120 min post-injection. The order of injection was counterbalanced across all 4 groups. Glucose was measured via a standard glucometer (CVS, MA, USA).

### Enzyme immunosorbent assays

Quantification of insulin (Crystal Chem, 90060; Downers Grove, IL, USA) and glucagon (Crystal Chem, 81519) was performed using commercially available EIA kits. Assays were performed according to the manufacturer’s suggestions. All data was analyzed using the Grubb’s test to identify outliers. For insulin, one statistical outlier was identified and removed from each of the experimental groups to allow for a normal distribution. For glucagon, one statistical outlier was identified and removed from the MORF1 CD group.

### Quantitative PCR

Total RNA was extracted using RNeasy (Qiagen) followed by cDNA conversion using RETROscript^®^. Real time PCR was performed on an ABI Prism 7700 (Applied Biosystems, Foster City, CA, USA). Taqman^®^ primers were purchased from Applied Biosystems (Plekhh2—Rn01769295; Gna12-Rn00667474_m1; Gapdh—Rn01775763_g1). Gapdh was used as the housekeeping gene based on preliminary analysis demonstrating similar expression across all groups. Final quantification of mRNA was obtained using the comparative cycle threshold (C_t_) method^56^ with data relative to SALF1 CD group.

### Statistical analysis

The results for each treatment are presented as mean ± S.E.M. and analyzed using a two-way analysis of variance (ANOVA) with F0 adolescent exposure (SALF1 vs. MORF1) and F1 diet (CD vs. FSD) as factors or three-way repeated measures ANOVA with time as the repeated factor. Pearson’s omnibus and Shapiro–Wilk *W* goodness-of-fit tests were used to test for normal distributions within data sets, and a Levene’s test was used to test for homogenous variances in all variables. The Grubb’s test was used to identify statistical outliers in sample sets that violated normality (only one outlier per group). Prism (version 8.0 GraphPad Software Inc., La Jolla, CA, USA) and SPSS (version 22 IBM SPSS, Armonk, NY, USA) statistical software were utilized for statistical analysis. A priori post hoc comparisons based on previous findings in FSD maintained animals^27^ were conducted using Student’s *t* tests with significance defined as *p* < 0.05.

### Transcriptome analyses

To examine differential gene expression and potential changes in gene and functional pathways, the liver was isolated and prepared for next generation sequencing using the Illumina platform (N = 6/treatment group). In brief, total RNA was extracted from a 5 mm^3^ liver (Qiagen) and 2 μg of total RNA was purified for mRNA. Isolated mRNA was further subjected to fragmentation, cDNA synthesis, adapter ligation, purification, and enrichment reactions (Illumina TruSeq Stranded mRNA sample preparation kit) to generate an equimolar cDNA library that underwent cluster generation in the Illumina sequencer. The RNA-seq generated 51-bp single end reads, and the quality of the fastq files was validated via FastQC. All RNA-seq samples displayed good quality scores, no adapter contamination, and good GC content. The reads were aligned to *Rattus norvegicus* 6.0 reference genome using STAR v2.5.3a based on Ensembl v92 annotation, and the counts were generated via STAR’s Gene-counts selection^57^. TMM normalization of sequencing data and differential gene expression analysis was performed in EdgeR^58^. Functional enrichment of Gene Ontology Biological Process, Molecular Function and Cellular Compartment terms in sets of differentially expressed genes were examined using g:Profiler selected for Rattus norvegicus with Benjamini–Hochberg FDR set at 0.05 and data sources restricted to manually annotated gene sets^26^.

## Supplementary Information

Supplementary Information 1.

Supplementary Information 2.

Supplementary Information 3.

Supplementary Information 4.

## References

[1] Kolodny A, Courtwright DT, Hwang CS, Kreiner P, Eadie JL, Clark TW, Alexander GC (2015). The prescription opioid and heroin crisis: A public health approach to an epidemic of addiction. Annu. Rev. Public Health..

[2] National Institute on Drug Abuse, University of Michigan. Institute for Social Research. Monitoring the future, national survey results on drug use. Bethesda, Md.: National Institute on Drug Abuse, U.S. Dept. of Health and Human Services, National Institutes of Health. Available from: Connect to full text http://WX3ZG9RE3E.search.serialssolutions.com/?V=1.0&L=WX3ZG9RE3E&S=JCs&C=MONITHEFUN&T=marc&tab=JOURNALS.

[3] Spencer MR, Weathers S (2020). Trends and risk factors of adolescent opioid abuse/misuse: understanding the opioid epidemic among adolescents. Int. J. Adolesc. Med. Health..

[4] Wehbeh L, Dobs AS (2020). Opioids and the hypothalamic–pituitary–gonadal (HPG) Axis. J. Clin. Endocrinol. Metab..

[5] Szklarczyk K, Korostynski M, Golda S, Piechota M, Ficek J, Przewlocki R (2016). Endogenous opioids regulate glucocorticoid-dependent stress-coping strategies in mice. Neuroscience.

[6] Goyal D, Limesand SW, Goyal R (2019). Epigenetic responses and the developmental origins of health and disease. J. Endocrinol..

[7] Brambilla F, Guerrini A, Guastalla A, Beretta P, Maio DD (1976). Glucose-insulin metabolism in heroin addicts. Neuropsychobiology..

[8] De Marinis L, Mancini A, Valle D, Bianchi A, De Luca AM, Fulghesu AM, Villa P, Mancuso S, Lanzone A (1997). Influence of chronic Naltrexone treatment on growth hormone and insulin secretion in obese subjects. Int. J. Obes. Relat. Metab. Disord..

[9] Ghodse AH (1977). Evaluation of blood glucose, insulin, growth hormone and cortisol response in heroin addicts. Pahlavi Med. J..

[10] Gosnell BA, Krahn DD (1993). The effects of continuous morphine infusion on diet selection and body weight. Physiol. Behav..

[11] Vuong C, Van Uum SH, O'Dell LE, Lutfy K, Friedman TC (2010). The effects of opioids and opioid analogs on animal and human endocrine systems. Endocr. Rev..

[12] Cicero TJ, Adams ML, Giordano A, Miller BT, O'Connor L, Nock B (1991). Influence of morphine exposure during adolescence on the sexual maturation of male rats and the development of their offspring. J. Pharmacol. Exp. Ther..

[13] Byrnes EM (2005). Chronic morphine exposure during puberty decreases postpartum prolactin secretion in adult female rats. Pharmacol. Biochem. Behav..

[14] Vassoler FM, Toorie AM, Teceno DN, Walia P, Moore DJ, Patton TD, Byrnes EM (2020). Paternal morphine exposure induces bidirectional effects on cocaine versus opioid self-administration. Neuropharmacology..

[15] Pachenari N, Azizi H, Semnaniann S (2019). Adolescent morphine exposure in male rats alters the electrophysiological properties of locus coeruleus neurons of the male offspring. Neuroscience..

[16] Moazen P, Azizi H, Salmanzadeh H, Semnanian S (2018). Adolescent morphine exposure induces immediate and long-term increases in impulsive behavior. Psychopharmacology (Berl)..

[17] Vassoler FM, Toorie AM, Byrnes EM (2019). Increased cocaine reward in offspring of females exposed to morphine during adolescence. Psychopharmacology (Berl)..

[18] Vassoler FM, Toorie AM, Byrnes EM (2018). Transgenerational blunting of morphine-induced corticosterone secretion is associated with dysregulated gene expression in male offspring. Brain Res..

[19] Vassoler FM, Oliver DJ, Wyse C, Blau A, Shtutman M, Turner JR, Byrnes EM (2017). Transgenerational attenuation of opioid self-administration as a consequence of adolescent morphine exposure. Neuropharmacology..

[20] Vassoler FM, Johnson-Collins NL, Carini LM, Byrnes EM (2014). Next generation effects of female adolescent morphine exposure: Sex-specific alterations in response to acute morphine emerge before puberty. Behav. Pharmacol..

[21] Byrnes JJ, Johnson NL, Carini LM, Byrnes EM (2013). Multigenerational effects of adolescent morphine exposure on dopamine D2 receptor function. Psychopharmacology (Berl)..

[22] Toorie AM, Vassoler FM, Qu FF, Schonhoff CM, Bradburn S, Murgatroyd CA, Slonim DK, Byrnes EM (2021). A history of opioid exposure in females increases the risk of metabolic disorders in their future male offspring. Addict. Biol..

[23] Zhan C (2018). POMC neurons: Feeding, energy metabolism, and beyond. Adv. Exp. Med. Biol..

[24] Ter Horst KW, Gilijamse PW, Ackermans MT, Soeters MR, Nieuwdorp M, Romijn JA, Serlie MJ (2016). Impaired insulin action in the liver, but not in adipose tissue or muscle, is a distinct metabolic feature of impaired fasting glucose in obese humans. Metabolism..

[25] Bock G, Chittilapilly E, Basu R, Toffolo G, Cobelli C, Chandramouli V, Landau BR, Rizza RA (2007). Contribution of hepatic and extrahepatic insulin resistance to the pathogenesis of impaired fasting glucose: role of increased rates of gluconeogenesis. Diabetes..

[26] Raudvere U, Kolberg L, Kuzmin I, Arak T, Adler P, Peterson H, Vilo J (2019). g:Profiler: a web server for functional enrichment analysis and conversions of gene lists (2019 update). Nucleic Acids Res..

[27] Toorie AM, Vassoler FM, Qu F, Schonhoff CM, Bradburn S, Murgatroyd CA, Slonim DK, Byrnes EM (2021). A history of opioid exposure in females increases the risk of metabolic disorders in their future male offspring. Addict Biol..

[28] Lu Y, Li Y, Sun Y, Ma S, Zhang K, Tang X, Chen A (2021). Differences in energy metabolism and mitochondrial redox status account for the differences in propensity for developing obesity in rats fed on high-fat diet. Food Sci Nutr..

[29] Abdul-Ghani M, DeFronzo RA (2007). Fasting hyperglycemia impairs glucose- but not insulin-mediated suppression of glucagon secretion. J. Clin. Endocrinol. Metab..

[30] Ruiter M, La Fleur SE, van Heijningen C, van der Vliet J, Kalsbeek A, Buijs RM (2003). The daily rhythm in plasma glucagon concentrations in the rat is modulated by the biological clock and by feeding behavior. Diabetes..

[31] Stern JH, Smith GI, Chen S, Unger RH, Klein S, Scherer PE (2019). Obesity dysregulates fasting-induced changes in glucagon secretion. J. Endocrinol..

[32] Gevi F, Fanelli G, Zolla L (2018). Metabolic patterns in insulin-resistant male hypogonadism. Cell Death Dis..

[33] Kanehisa M, Goto S (2000). KEGG: Kyoto encyclopedia of genes and genomes. Nucleic Acids Res..

[34] Degli Esposti D, Hamelin J, Bosselut N, Saffroy R, Sebagh M, Pommier A, Martel C, Lemoine A (2012). Mitochondrial roles and cytoprotection in chronic liver injury. Biochem. Res. Int..

[35] Yang S, Kwak S, Lee JH, Kang S, Lee SP (2019). Nonalcoholic fatty liver disease is an early predictor of metabolic diseases in a metabolically healthy population. PLoS One..

[36] Lecat S, Matthes HW, Pepperkok R, Simpson JC, Galzi JL (2015). A fluorescent live imaging screening assay based on translocation criteria identifies novel cytoplasmic proteins implicated in G protein-coupled receptor signaling pathways. Mol. Cell Proteomics..

[37] Chang SW, McDonough CW, Gong Y, Johnson TA, Tsunoda T, Gamazon ER, Perera MA, Takahashi A, Tanaka T, Kubo M, Pepine CJ, Johnson JA, Cooper-DeHoff RM (2018). Genome-wide association study identifies pharmacogenomic loci linked with specific antihypertensive drug treatment and new-onset diabetes. Pharmacogenomics J..

[38] Chisholm KW, O'Dea K (1987). Effect of short-term consumption of a high fat diet on glucose tolerance and insulin sensitivity in the rat. J. Nutr. Sci. Vitaminol. (Tokyo)..

[39] Tian X, Zhang Y, Li H, Li Y, Wang N, Zhang W, Ma B (2020). Palmatine ameliorates high fat diet induced impaired glucose tolerance. Biol. Res..

[40] Lukito W, Wibowo L, Wahlqvist ML (2019). Maternal contributors to intergenerational nutrition, health, and well-being: Revisiting the Tanjungsari Cohort Study for effective policy and action in Indonesia. Asia Pac. J. Clin. Nutr..

[41] Muhlhausler BS, Gugusheff JR, Ong ZY, Vithayathil MA (2013). Nutritional approaches to breaking the intergenerational cycle of obesity. Can. J. Physiol. Pharmacol..

[42] Herring SJ, Oken E (2011). Obesity and diabetes in mothers and their children: Can we stop the intergenerational cycle?. Curr. Diab, Rep..

[43] Reusens B, Remacle C (2001). Intergenerational effect of an adverse intrauterine environment on perturbation of glucose metabolism. Twin Res..

[44] Priante E, Verlato G, Giordano G, Stocchero M, Visentin S, Mardegan V, Baraldi E (2019). Intrauterine growth restriction: new insight from the metabolomic approach. Metabolites..

[45] Perez MF, Lehner B (2019). Intergenerational and transgenerational epigenetic inheritance in animals. Nat. Cell Biol..

[46] Chavey A, Ah Kioon MD, Bailbe D, Movassat J, Portha B (2014). Maternal diabetes, programming of beta-cell disorders and intergenerational risk of type 2 diabetes. Diabetes Metab..

[47] Patti ME (2013). Intergenerational programming of metabolic disease: Evidence from human populations and experimental animal models. Cell Mol. Life Sci..

[48] Heindel JJ, Blumberg B (2019). Environmental obesogens: Mechanisms and controversies. Annu. Rev. Pharmacol. Toxicol..

[49] Chamorro-Garcia R, Sahu M, Abbey RJ, Laude J, Pham N, Blumberg B (2013). Transgenerational inheritance of increased fat depot size, stem cell reprogramming, and hepatic steatosis elicited by prenatal exposure to the obesogen tributyltin in mice. Environ. Health Perspect..

[50] Browne CJ, Godino A, Salery M, Nestler EJ (2020). Epigenetic mechanisms of opioid addiction. Biol. Psychiatry..

[51] Bodi CM, Vassoler FM, Byrnes EM (2016). Adolescent experience affects postnatal ultrasonic vocalizations and gene expression in future offspring. Dev. Psychobiol..

[52] Johnson NL, Carini L, Schenk ME, Stewart M, Byrnes EM (2011). Adolescent opiate exposure in the female rat induces subtle alterations in maternal care and transgenerational effects on play behavior. Front. Psychiatry..

[53] Sato K, Sato M (2017). Multiple ways to prevent transmission of paternal mitochondrial DNA for maternal inheritance in animals. J. Biochem..

[54] Feng YM, Jia YF, Su LY, Wang D, Lv L, Xu L, Yao YG (2013). Decreased mitochondrial DNA copy number in the hippocampus and peripheral blood during opiate addiction is mediated by autophagy and can be salvaged by melatonin. Autophagy..

[55] Spear LP (2004). Adolescent brain development and animal models. Ann. N. Y. Acad. Sci..

[56] Pfaffl MW (2001). A new mathematical model for relative quantification in real-time RT-PCR. Nucleic Acids Res..

[57] Dobin A, Davis CA, Schlesinger F, Drenkow J, Zaleski C, Jha S, Batut P, Chaisson M, Gingeras TR (2013). STAR: ultrafast universal RNA-seq aligner. Bioinformatics..

[58] Robinson MD, McCarthy DJ, Smyth GK (2010). edgeR: a Bioconductor package for differential expression analysis of digital gene expression data. Bioinformatics..

