# Supplementary material for: Intergenerational effects of preconception opioids on glucose homeostasis and hepatic transcription in adult male rats
Source: Sci Rep. 2022 Jan 31;12:1599. doi: 10.1038/s41598-022-05528-w (PMC8803846; doi:10.1038/s41598-022-05528-w)
Supplement: Supplementary file 4 — Supplementary Information 4. [file 41598_2022_5528_MOESM4_ESM.docx]

Supplemental Table 1

*Differentially Expressed Genes*

Complete list of significant differentially expressed genes as a function of F0 exposure or diet condition.

Supplemental Table 2

*Gene Ontology (GO) Terms*

List of GO terms significantly affected by F0 exposure or diet condition.

Supplemental Table 3

KEGG Pathways

List of KEGG pathways significantly affected by F0 exposure in CD maintained males or by diet condition in SALF1 males.
